# Supplementary material for: Context-dependent interaction between goal-directed and habitual control under time pressure
Source: Commun Psychol. 2026 May 18;4:88. doi: 10.1038/s44271-026-00455-2 (PMC13222367; doi:10.1038/s44271-026-00455-2)
Supplement: Supplementary file 2 — Supplementary_Information [file 44271_2026_455_MOESM2_ESM.pdf]

Context-Dependent Interaction Between Goal-Directed and Habitual Control Under Time Pressure - Supplementary Material

Ben J. Wagner [1,4,*,+], Sascha Frölich [1,+], Sarah Schwöbel [1], Michael N. Smolka [2], Stefan J. Kiebel [1,3]

[1]Department of Psychology, Technische Universität Dresden, Dresden, Germany
[2]Department of Psychiatry and Psychotherapy, Technische Universität Dresden, Dresden, Germany
[3]Centre for Tactile Internet with Human-in-the-Loop
[4]Department of Psychiatry and Psychotherapy, University of Tuebingen, Tuebingen, Germany
+ these Authors contributed equally
*ben.jonathan.wagner@gmail.com

March 25, 2026

I. Methods

i. The Action Sequence Task

i.1 Criterion Test and Test Trials

After the instructions and before the main experiment, participants were told that their understanding of the task will be tested by a short test. In the test, participants were presented 13 successive dual-target trials. Participants were explicitly told that there is no time-limit during these test trials, and that they should always choose the option with the higher reward probability when possible. Participants were further told the stimulus positions with the higher reward probabilities. If a participant chose a low-reward-probability option in more than one of the 13 trials, they were again instructed about the test and their task. If failed, participants could repeat the test a maximum of two times, resulting in a maximum of three possible iterations. If a participant failed all three iterations, they were excluded from further participation. After successful completion of the criterion test, participants performed 20 test trials to get used to the pace of the experiment.

i.2 Counterbalancing

To account for hemispheric effects as well as for learning effects, participants were randomly assigned to one of four different counterbalancing groups, as shown in table S1.

Mirroring. Mirrored counterbalancing groups were introduced to account for hemispheric effects, like for instance a preference for the right hand (which is the dominant hand for the majority of the population). In reward contingency 1, the stimulus positions at the top left and bottom right had reward probabilities of 80%, while bottom left and top right had 20%. These were reversed in reward contingency 2, essentially mirroring the rewards along the vertical. Sequence 2 was a mirrored version of sequence 1, mirrored along the vertical, so if a stimulus appeared at the top

left in sequence 1, it appeared at the top right in sequence 2. The stimuli in dual-target trials were mirrored in the same way for counterbalancing. Mirroring both stimuli and rewards ensured that congruent (incongruent) trials remained congruent (incongruent) even after mirroring.
Reversed block orders. Reversal of block orders was done to account for learning effects (Table S1). Half of participants started with the random condition on day one and the repeating condition on day two, while the other half started with the repeating condition on day one and the random condition on day two. If all participants started for instance with the random condition on day one, reduced reaction times and error rates in the repeating condition (see main manuscript) could not be distinguished from possible learning effects at the beginning of the experiment.

i.3 Difference to previously published Task Version

The task paradigm used for this study was a revised and refined version of the previously published AST (Frölich et al., 2023). In the previously published version of the AST, some DTT trials featured two stimuli with the same reward probability (*Neutral DTTs*, with both stimuli either 20% or both 80% reward probability). For the present paradigm, we limited DTT types to random, congruent, and incongruent, to have one goal-directed response option in every DTT. Furthermore, in the previously published task version (Frölich et al., 2023), feedback consisted of a euro coin in the case of a point reward, and a white dot in the case of no point reward. To make feedback for both outcomes more similar, in the present version, feedback consists of a green smiley (point reward) and a red frowney (no point reward). The proportion of DTTs varied around 15% in the previous AST. In the present version, each block of 480 trials contains exactly 72 (15%) DTTs per block, with exactly 36 incongruent and 36 congruent DTTs in the repeating sequence condition. Lastly, in the previous version of the AST, stimulus sequences for the random condition were created pseudo-randomly subject to some constraints, and continuously for a whole block of 480 trials. In the present version, stimulus sequences for the random condition consisted of concatenated sequences of 12 elements, where each such sequence was created subject to the same constraints as the sequence for the Rep condition. Concatenation was performed such that no stimulus is repeated twice in a row.

i.4 Participants

Participants were randomly assigned to one of the 4 counterbalancing groups. Of 175 participants who initiated the experiment, 25 (14.3%) did not pass the Criterion Test. 3 participants timed out, 3 were excluded for initiating the study twice, 1 could not start part 2 of the experiment due to a user error, and 1 participant had technical problems during execution of the experiment. 10 participants could not finish the study due to a user error on the researchers' side, and 10 because of technical problems with the recruitment platform. 41 participants did not finish the experiment due to unknown reasons. 81 participants completed the experiment on both days. Of those 81 participants, 13 were excluded because they did not finish both parts of the experiment at approximately the same time of day or on two consecutive days, and 3 were excluded because of large error rates (error rates of $> 15\%$ in either single-target trials or dual-target trials or both). This resulted in 65 participants eligible for data analysis, (16 in counterbalancing group 1, 15 in group 2, 18 in group 3, 16 in group 4). In order for each counterbalancing group to contain the same number of participants for data analysis, we randomly excluded 1 participant from group 1, 3 from group 3, and 1 from group 4. This resulted in 15 participants in each of the four groups.

Table S1: The four different counterbalancing groups.

	**Sequence 1 with Reward Contingency 1**	**Sequence 2 with Reward Contingency 2**
**Block Order 1**	Group 1	Group 3
**Block Order 2**	Group 2	Group 4

Table S2: The two different block orders.

	**Block order 1**	**Block order 2**
Day 1	Rep	Rand
	Rand	Rep
	Rep	Rand
	Rand	Rep
	Rep	Rand
	Rand	Rep
Day 2	Rand	Rep
	Rep	Rand
	Rand	Rep
	Rep	Rand
	Rand	Rep
	Rep	Rand
	Rand	Rep
	Rep	Rand

i.5 Sequence Generation

Stimulus sequences were generated as sequences of 12 elements. Such 12-element sequences was generated in such a way that the same stimulus position did not appear twice in a row, and such that a certain stimulus position followed another stimulus position only once in the sequence (so for instance 1-2-1-2-... was inadmissible). Each 12-element sequence contained each of the four stimulus positions exactly three times. For each block (480 trials) of the Random-Sequence condition, 40 different 12-element sequences were randomly created and concatenated such that no repeating stimulus positions occurred. 72 Dual-target trials were inserted pseudo-randomly into each block of 480 trials, such that DTTs are separated by two STTs at least and nine STTs at most, and in a way that the distribution of joker types and separations between jokers were roughly uniformly distributed.

ii. Generative Modeling

ii.1 Drift-Diffusion Modeling

Model fitting. For the drift-diffusion model, fitting was performed using the HSSM toolbox Fengler et al. (prep). Model estimation was done in Python (Version 3.11.9). For group-level means we used uniform priors defined over numerically plausible parameter ranges (see code and data availability section for details). Model-fitting was done using MCMC sampling. Chain convergence was assessed via the Gelman-Rubinstein convergence diagnostic $\hat{R}$ and sampling was continued until $1 \leq \hat{R} \leq 1.05$ for all group-level and individual-subject parameters. DDM was fitted with collapsing bounds to simulate time pressure, and lapse probabilities, that is, the probability of a random choice.

II. Results

i. RTs and reward location

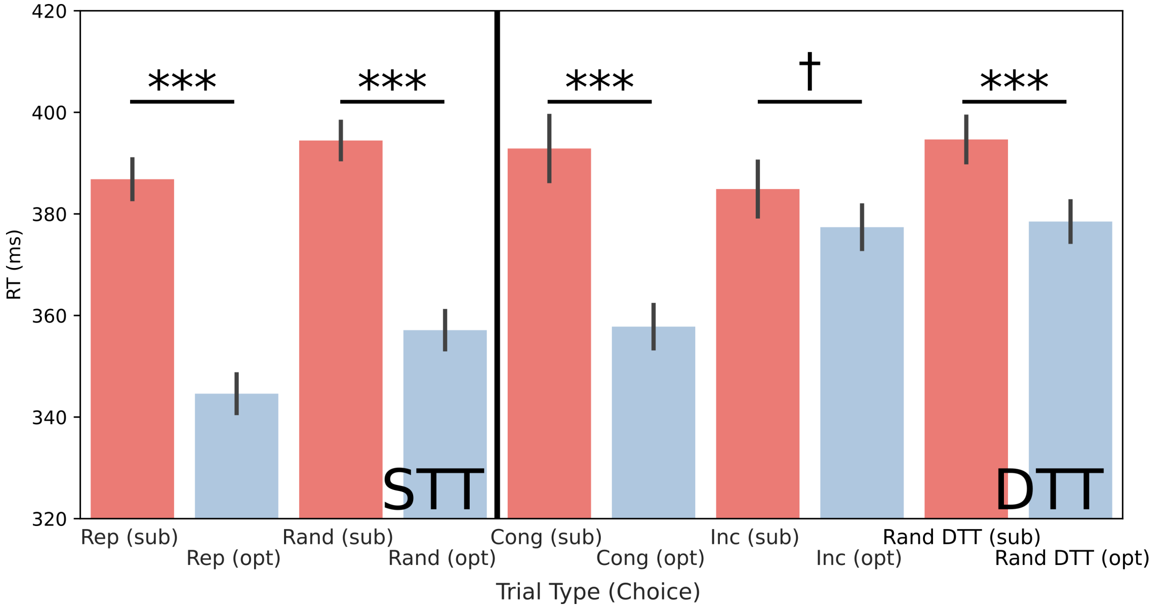

Figure S1: Left panel: Reaction times in single-target trials (STTs) as a function of reward location (high/opt vs. low/sub reward probability) **Right panel**: Reaction times in dual-target trials (DTTs) as a function of reward location (high/opt vs. low/sub reward probability).

ii. Generative Modeling and Model Comparison

Fig. S2 shows the subject-level posterior parameter distributions for the winning model (model 3). Fig. S3 shows the changes in posterior means of parameters from day 1 to day 2. Table S3 shows subject-level WAIC values and Bayes Factors for model comparison.

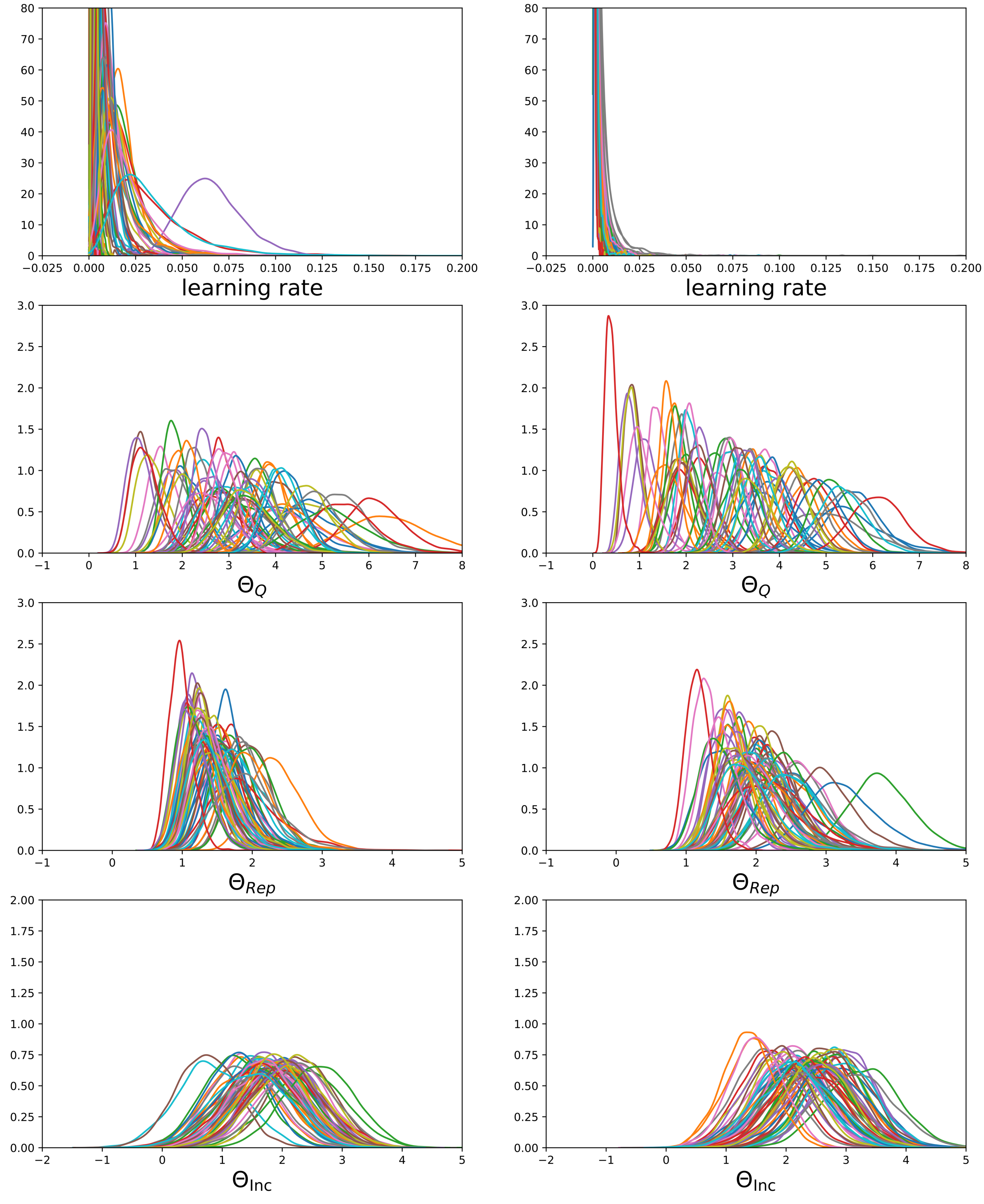

Figure S2: Posterior Distributions of inferred parameters of the best-performing model M 3. Left panel: distributions on day 1. Right panel: distributions on day 2

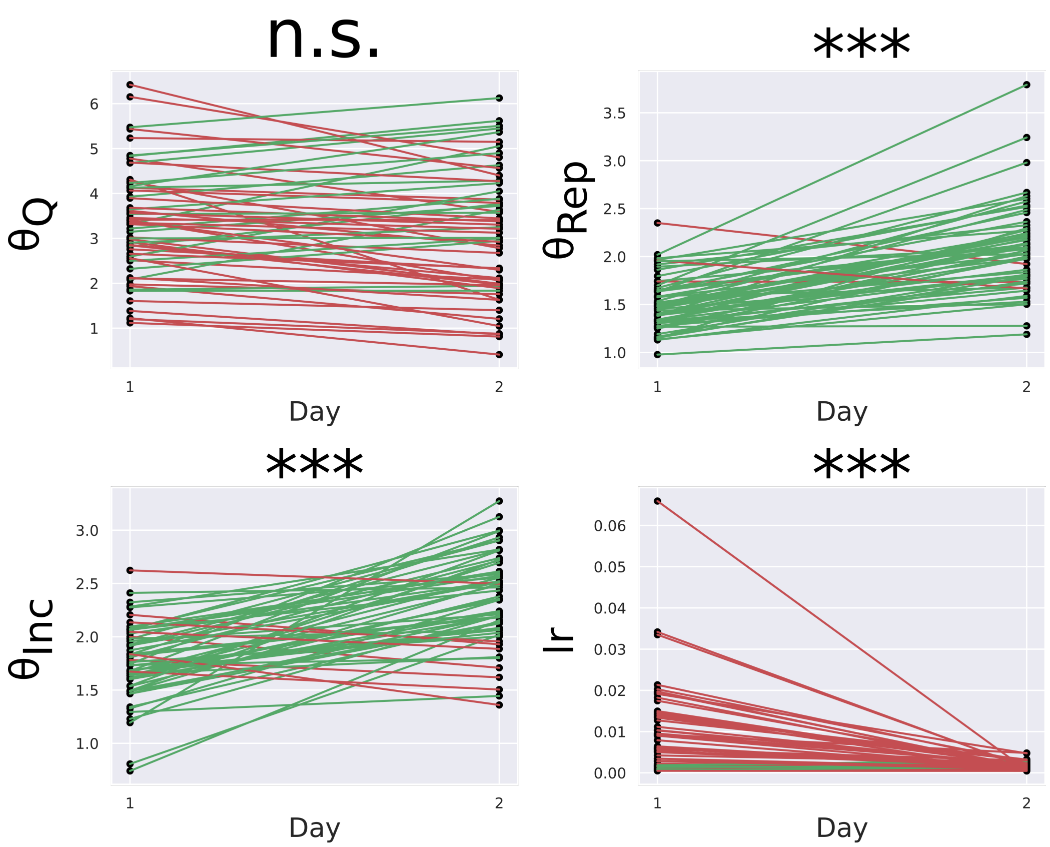

Figure S3: Changes of posterior means from day 1 to day 2. Learning rate, θ_{Rep}, and θ_{Switch} increases significantly from day 1 to day 2 (paired two-sample t-tests). $***: p < 0.001, **: p < 0.01, *: p < 0.05$.

iii. Drift-Diffusion Modeling

Fig. S4 shows the posterior distributions of drift-diffusion parameters on day one and two.

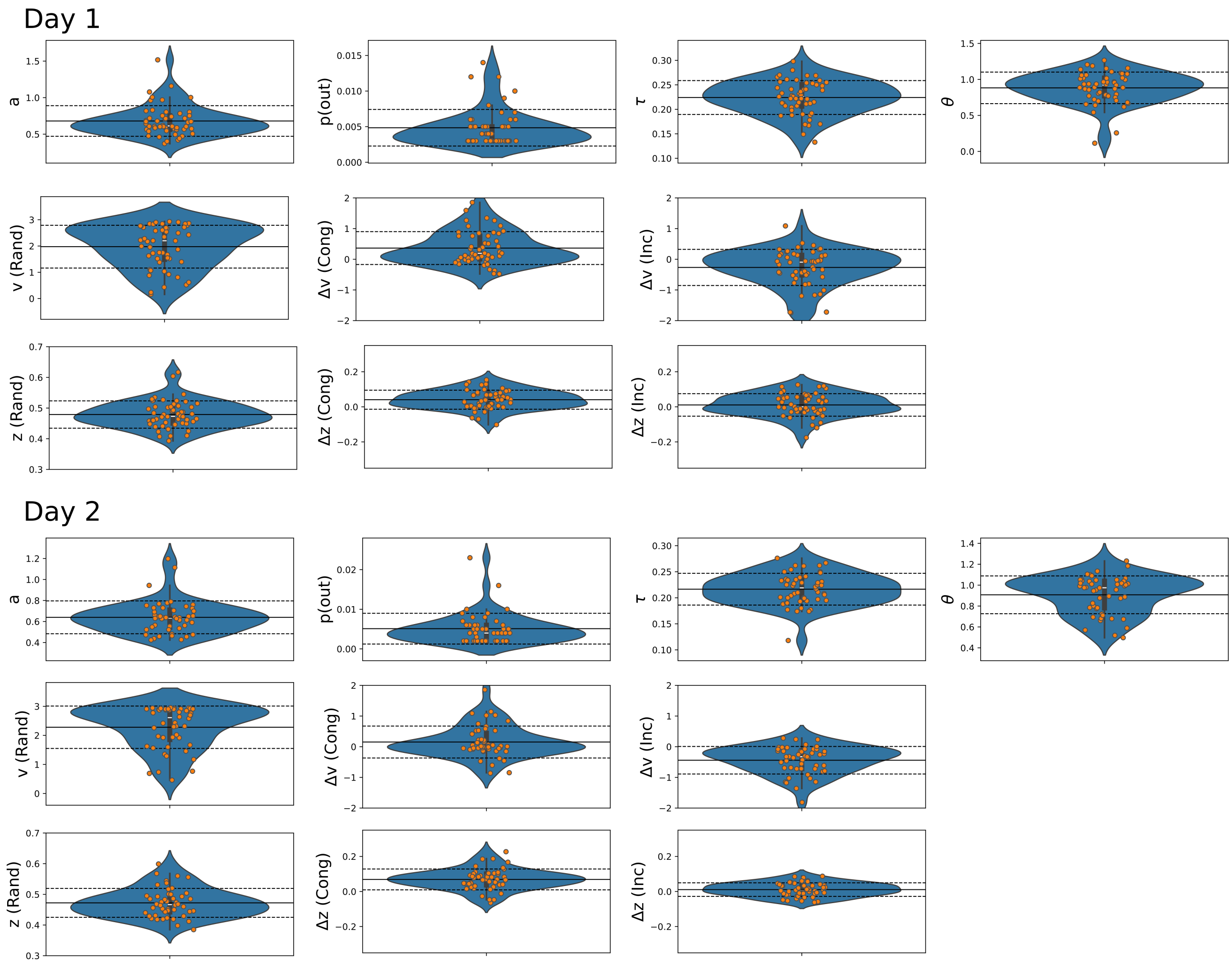

Figure S4: DDM Results: Means of posterior distributions for individual participants. *a* Boundary separation, *p(out)* probability of a random choice, τ non-decision time (in seconds), θ boundary angle (in radians), *v (Rand)* drift rate in random DTTs, Δ *(Cong)* Difference of drift rate in congruent DTTs compared to random DTTs, Δ *(Inc)* Difference of drift rate in incongruent DTTs compared to random DTTs, *z (Rand)* starting point bias in random DTTs, Δ *(Cong)* Difference of starting point bias in congruent DTTs compared to random DTTs, Δ *(Inc)* Difference of starting point bias in incongruent DTTs compared to random DTTs.

iv. Posterior Predictive Checks

We performed posterior predictive checks to assess whether the best-fitting model could reproduce key empirical patterns in goal-directed responding across task contexts. We therefore used the posterior draws from the fitted hierarchical model to simulate binary goal-directed choices on DTT for each participant. We then aggregated simulated choices in the same way as the observed data, computing the proportion of optimal (goal-directed) choices separately for each DTT type (random, congruent, incongruent) and directly compare them to the observed group-level proportions (see Fig. S5 below).

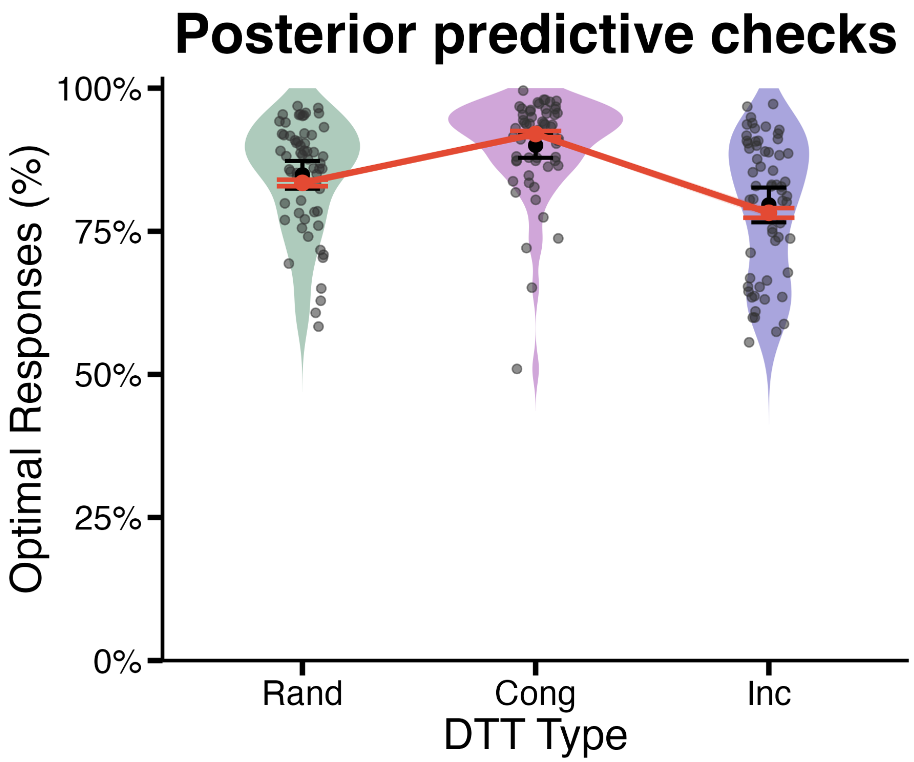

Figure S5: Posterior Predictive Checks: Group-level posterior predictive checks for the winning model 3. Violin plots and black points show the empirical percentage of optimal responses for each participant across the three DTT contexts (Rand, Cong, Inc). Black horizontal bars represent the empirical group means with their SE. Red points and red connecting lines show the posterior predictive mean estimates and their SE obtained by simulating choices from the posterior distribution of model 3.

v. Parameter Recovery

We performed parameter recovery for the best-fitting model (model 3). During MCMC, we generated posterior-predictive datasets by simulating DTT choice datasets conditional on joint post-warm-up parameter draws, across the whole posterior distribution while preserving the original trial structure, outcomes, and all regressors (subject-level learning-rate (lr) and weights θ_Q, θ_{Rep}, θ_{Inc}, each estimated separately for Day 1 and Day 2). Across this posterior-predictive pool, we randomly sampled K = 15 synthetic datasets and refit each dataset with the same hierarchical implementation as in the main analysis. Parameter recovery was assessed by computing Pearson correlations between the known generating parameter values (from the joint posterior draw used to simulate each dataset) and the recovered parameter estimates (posterior means), computed separately for each parameter and day and pooled across subjects and simulated datasets (see Fig. S6).

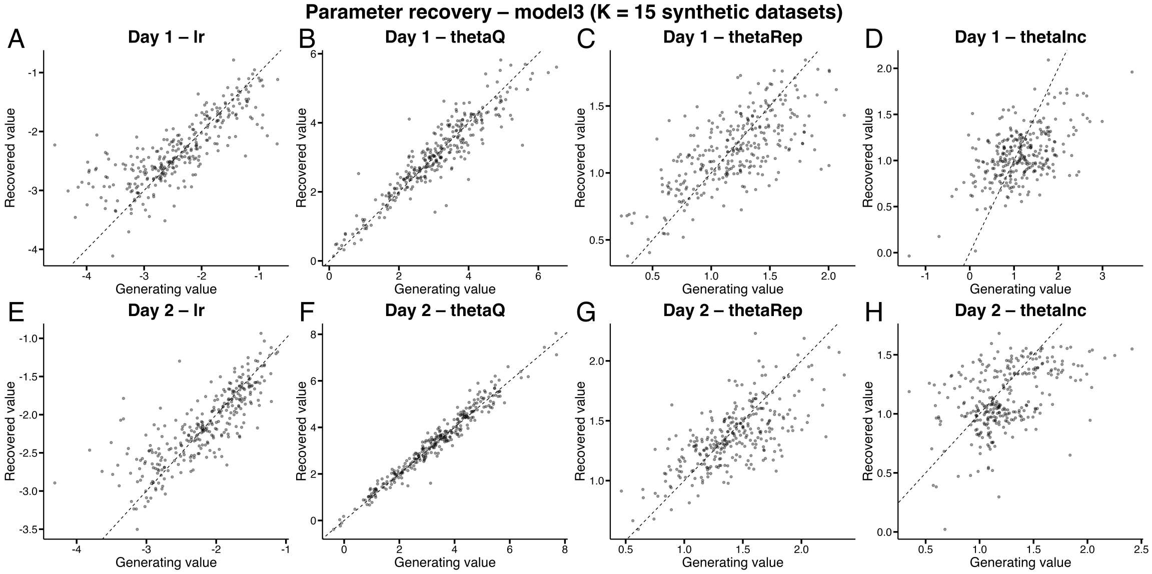

Figure S6: Parameter Recovery for model 3: Parameter recovery for model 3 across two days, with panels A–D showing Day 1 and panels E–H showing Day 2. Each panel corresponds to one model parameter (A and E: learning rate (lr), B and F: θ_Q, C and G: θ_{Rep} D and H: θ_{Inc}) and plots generating parameter values drawn from the joint posterior against the recovered posterior means obtained after refitting the full JAGS model to synthetic datasets. Note that because simulating directly from the posterior we account for the whole within and between subject variance-covariance structure. Scatter plots of true generating values (x-axis) against posterior means after refitting (y-axis) for each parameter of model 3. Each facet corresponds to one parameter. Points represent individual participants across 15 simulated full datasets. Parameters showed good recovery for day1, lr: r = 0.80, : θ_Q r = 0.92 , θ_{Rep}: r = 0.69, θ_{Inc}: r = 0.53 and day2, lr: r = 0.81, : θ_Q r = 0.98 , θ_{Rep}: r = 0.69, θ_{Inc}: r = 0.47.

vi. Hyperparameter distributions

We here show hyperparameter distributions for model 3 for all parameters for day 1 and day 2 as well as their difference. Fig. S9 shows the distributions of posterior means for drift-diffusion parameters on Day 1 and Day 2, as well as their differences.

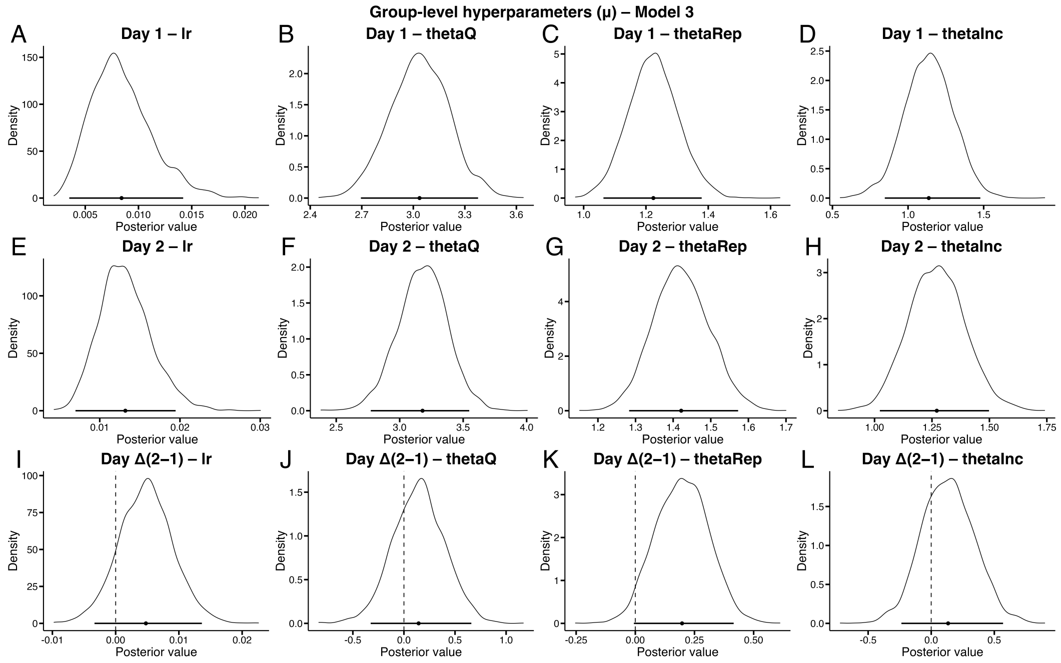

Figure S7: Hyperparameter distributions: Posterior hyperparameter distributions. Top row: Hyperparameter distributions for day 1. A: learning rate (lr), B: θ_Q, C: θ_{Rep}, D: θ_{Inc}. Middle row: Corresponding posterior distributions for day 2. E: lr, F: θ_Q, G: θ_{Rep}, H: θ_{Inc}. Bottom row: Posterior difference distributions (day 2 – day 1). Positive values indicate higher parameter values on day 2. (I) Δlr, $\text{BF}_{10}(\text{day 2} > \text{Day 1}) = 7.01$. J: $\Delta\theta_Q$, $\text{BF}_{10} = 2.55$. K: $\Delta\theta_{\text{Rep}}$, $\text{BF}_{10} = 29.5$. (L) $\Delta\theta_{\text{Inc}}$, $\text{BF}_{10} = 2.71$.

vii. Disentangling effects of reward location and task context

To explicitly disentangle reward-location effects from context effects (congruent vs. incongruent action sequence) on RTs we conducted a set of control analyses. Given that there was only one option in SSTs all analyses were performed using linear mixed-effects models with random effects at the participant level, thereby accounting for individual differences in baseline RTs, sensitivity to reward location and task context. First, we tested whether block (Random vs Repetition) effects could be explained by reward location alone. We therefore fit a mixed-effects model predicting STT RTs from block condition (Random vs Repetition), participant-specific reward location (High vs Low), and their interaction:

$$RT_{\text{STT}} \sim \texttt{blockcondition} \times \texttt{rewardloc} + (1 + \texttt{blockcondition} + \texttt{rewardloc} \,|\, \texttt{participant}) \quad (1)$$

This analysis revealed a main effect of reward location ($\beta = +37.5$ ms, SE = 1.96, $t(59.98) = 19.18$, $p < .001$), with responses to low-reward locations being significantly slower than to high-reward locations. Importantly, we also observed a robust main effect of block condition, with repetition blocks producing faster RTs than random blocks even when accounting for reward location ($\beta = -12.1$ ms, SE = 0.97, $t(59.99) = -11.54$, $p < .001$). We additionally found a significant interaction indicating that reward-location effects were modestly amplified in repetition blocks ($\beta = +4.68$ ms, SE = 0.70, $t(60.11) = 6.66$, $p < .001$). Critically, repetition blocks (combined congruent and incongruent contexts) were faster than random blocks at both high- and low-reward locations, demonstrating that repetition effects cannot be reduced to reward-location alone. We next looked at dual-target trials (DTTs) to test whether congruency effects in the repetition block could be explained by individual differences in reward location sensitivity. To this end, we computed a participant-specific reward sensitivity measure (ΔSTT) defined as the difference in STT RTs between low- and high-reward locations, and included this measure as a between-participant covariate in a mixed-effects model predicting DTT RTs:

$$RT_{\text{DTT}} \sim \texttt{dtt\_type} + \Delta STT_{\text{rewardloc}} + \texttt{dtt\_type} \times \Delta STT_{\text{rewardloc}} + (1 + \texttt{dtt\_type} \,|\, \texttt{participant}) \quad (2)$$

This analysis revealed a main effect of DTT type such that congruent trials were significantly faster than random trials ($\beta = -17.18$ ms, SE = 4.80, $t(60.19) = -3.58$, $p < .001$). In contrast, incongruent trials did not differ reliably from random trials in RT ($\beta = -2.14$ ms, SE = 3.20, $t(60.91) = -0.67$, $p = .506$). Crucially, individual reward sensitivity (ΔSTT) did not significantly predict DTT RTs ($\beta = -0.40$, SE = 0.24, $t(60.01) = -1.64$, $p = .107$), nor did it interact with DTT type (both interaction terms $p > .62$). Thus, participants who showed stronger reward-location invigoration in STTs did not show larger congruency effects in DTTs. Together, these results indicate that the congruency-related RT advantage in DTTs cannot be explained by individual differences in reward sensitivity measured in STTs. Finally, we conducted an additional control analysis that accounted for trialwise reward location in DTTs (i.e., whether the executed action entered a high- vs. low-reward location), while still controlling for participant-level reward sensitivity:

$$RT_{\text{DTT}} \sim \texttt{dtt\_type} + \Delta STT_{\text{rewardloc}} + \texttt{dtt\_type} \times \Delta STT_{\text{rewardloc}} + (1 + \texttt{dtt\_type} \,|\, \texttt{participant}) \quad (3)$$

This analysis confirmed a robust trialwise reward-location effect, with responses entering low-reward locations being significantly slower than those entering high-reward locations ($\beta = +11.62$ ms, SE = 0.85, $t(57{,}955) = 13.62$, $p < .001$). Importantly, congruency effects remained robust after controlling for both trialwise reward location and individual reward sensitivity: congruent DTTs were substantially faster than random DTTs ($\beta = -18.77$ ms, SE = 1.62, $t(60.03) = -11.60$, $p < .001$). Moreover, a direct pairwise contrast between incongruent and congruent DTTs revealed

Table S3: Individual participant WAIC scores and approximate-Bayes Factors.

Subject ID	WAIC (M1)	WAIC (M2)	BF (M2 vs M1)	WAIC (M3)	BF (M3 vs M1)
1	665.35	664.59	1.47	658.04	38.79
2	983.74	983.27	1.26	986.63	0.24
3	598.79	595.93	4.19	594.53	8.41
4	949.27	950.61	0.51	946.82	3.41
5	1184.47	1182.85	2.25	1171.70	592.60
6	1124.07	1107.92	3223.22	1098.84	301422.86
7	648.81	644.47	8.79	643.01	18.15
8	1119.19	1121.83	0.27	1115.15	7.51
9	476.94	475.86	1.72	476.56	1.21
10	832.39	826.80	16.40	818.40	1094.69
11	342.97	344.05	0.58	343.56	0.75
12	481.87	483.54	0.43	483.56	0.43
13	1004.77	1002.54	3.05	997.85	31.84
14	629.12	630.79	0.43	629.53	0.81
15	1282.15	1275.26	31.29	1261.16	36055.59
16	1047.71	1045.19	3.53	1039.04	76.17
17	796.50	794.22	3.12	790.86	16.74
18	614.63	614.04	1.35	612.70	2.63
19	1237.42	1226.36	251.66	1219.64	7251.18
20	527.47	528.03	0.75	528.89	0.49
21	938.79	939.87	0.58	935.92	4.22
22	1064.54	1066.24	0.43	1063.02	2.13
23	903.02	893.78	101.36	884.15	12476.00
24	968.55	966.69	2.54	958.31	167.21
25	713.61	712.50	1.74	712.37	1.86
26	1006.90	1003.43	5.69	1000.47	24.90
27	577.90	578.35	0.80	576.52	2.00
28	763.57	763.64	0.97	762.22	1.96
29	1072.09	1073.78	0.43	1070.96	1.76
30	679.45	678.61	1.52	677.66	2.45
31	497.82	499.51	0.43	497.63	1.10
32	377.61	375.97	2.27	374.69	4.32
33	819.92	817.44	3.45	811.47	68.25
34	261.70	259.85	2.52	258.67	4.54
35	774.09	774.28	0.91	776.52	0.30
36	469.43	470.82	0.50	472.23	0.25
37	1164.72	1167.40	0.26	1164.33	1.21
38	333.31	329.28	7.51	326.73	26.90
39	531.09	531.74	0.72	531.87	0.68
40	708.35	704.43	7.11	700.96	40.21
41	568.61	563.98	10.13	563.75	11.36
42	485.54	482.14	5.49	480.11	15.12
43	357.69	359.27	0.45	361.48	0.15
44	242.43	241.19	1.86	240.38	2.79
45	829.61	830.56	0.62	831.70	0.35
46	802.99	798.19	11.01	790.81	440.12
47	703.95	702.72	1.85	701.70	3.09
48	576.96	570.30	27.95	566.03	235.45
49	368.30	367.69	1.36	368.93	0.73
50	573.52	573.86	0.84	571.99	2.15
51	393.52	393.78	0.88	392.87	1.39
52	978.55	981.45	0.23	982.58	0.13
53	711.78	709.07	3.86	707.83	7.21
54	1275.03	1275.19	0.92	1270.10	11.76
55	609.82	601.01	81.81	598.83	243.35
56	682.57	684.13	0.46	682.76	0.91
57	832.01	835.39	0.19	836.20	0.12
58	273.63	272.64	1.64	271.86	2.43
59	661.61	660.27	1.96	659.57	2.78
60	341.04	342.35	0.52	343.81	0.25

slowing for incongruent relative to congruent trials (Inc − Cong: $\beta = +15.45$ ms, SE = 1.47, $t(61.4) = 10.47$, $p < .001$). By contrast, the participant-level reward sensitivity covariate ΔSTT did not significantly predict DTT RTs ($\beta = -0.35$ ms, SE = 0.24, $t(60.04) = -1.44$, $p = .16$). Taken together reward location does influence response times, however, repetition effects in STTs and congruency effects in DTTs remained significant after controlling for reward location at both the trial and participant levels. We interpret these results demonstrate that the reported congruency effects cannot be explained solely by differences in expected reward and instead reflect context-dependent interference or facilitation between the acquired action sequence and goal-directed choice.

viii. Context-dependent change in DDM parameters

We found that changes in drift rate and response bias are indeed related: individual differences in the shift from the random context to both the congruent and incongruent contexts showed moderate negative correlations (congruent: $\rho = -0.36$; incongruent: $\rho = -0.46$). Second, we observed a significant starting-point (bias) shift from the random to the congruent context (see Supplementary Figure S9 and Figure 6 in the main manuscript), whereas the corresponding bias shift from random to incongruent was not significant. In the congruent context, participants exhibited a bias toward the optimal response, which is theoretically consistent with an automatic, habit-aligned response tendency when habitual and goal-directed actions coincide (i.e., no response conflict). Third, examining individual parameter changes revealed a qualitative dissociation between contexts. While most participants ($\sim$ 40) showed evidence for a reduction in drift rate when transitioning from the random to the incongruent context (see Supplementary Figure S9A), most participants ($\sim$ 40) did not show a substantial drift-rate change from the random to the congruent context (see the x-axis in Supplementary Figure S9B). Instead, the congruent context primarily affected response bias. Together, this pattern suggests that congruent trials are characterized by proactive, bias-based facilitation of the habitual response, consistent with automatic processing. In contrast, incongruent trials primarily affect evidence accumulation, reflected in reduced drift rates, consistent with response conflict at the level of evidence accumulation.

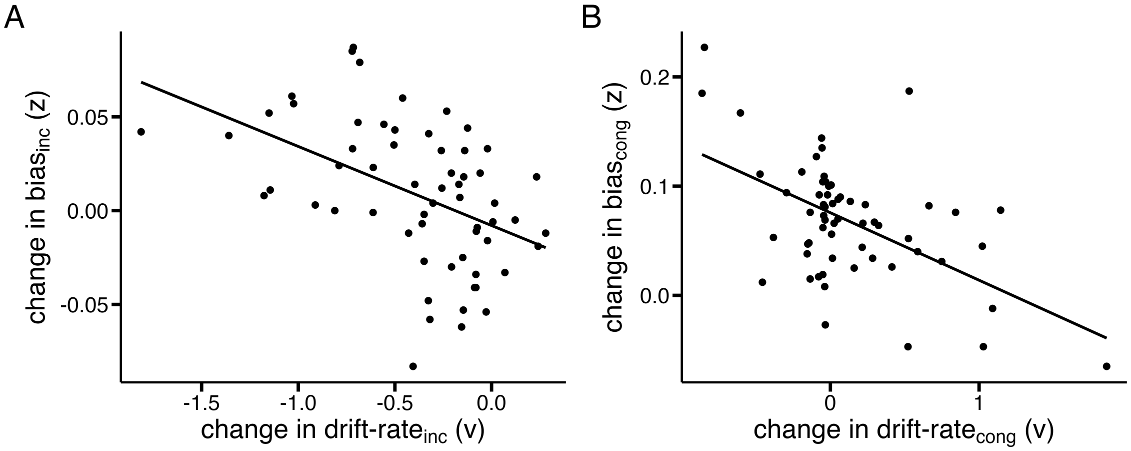

Figure S8: Relation between DDM parameters. Posterior hyperparameter distributions. **A:** Association between changes in drift rate (Δv) and response bias (Δz) from the random to the incongruent context. **B:** Association between changes in drift rate (Δv) and response bias (Δz) from the random to the congruent context.

ix. Analysis of all 65 participants that completed the task

Our initial goal was to ensure a fully balanced task design across our four experimental groups. After excluding participants who did not complete the task or failed to meet our inclusion criteria, we were left with 65 eligible participants (16 in Group 1, 15 in Group 2, 18 in Group 3, and 16 in Group 4). Because Group 2 had the lowest number of participants (15), we balanced the final sample by including exactly 15 participants per group, resulting in the 60 participants reported in the main manuscript. For transparency, we re-ran our analyses on the full sample of 65 participants to confirm that our results were not driven by this balancing procedure. Below, we first present the complete model comparison (WAIC values) and the hyperparameter distributions for the winning model (Model 3), followed by the behavioral analyses for this extended sample.

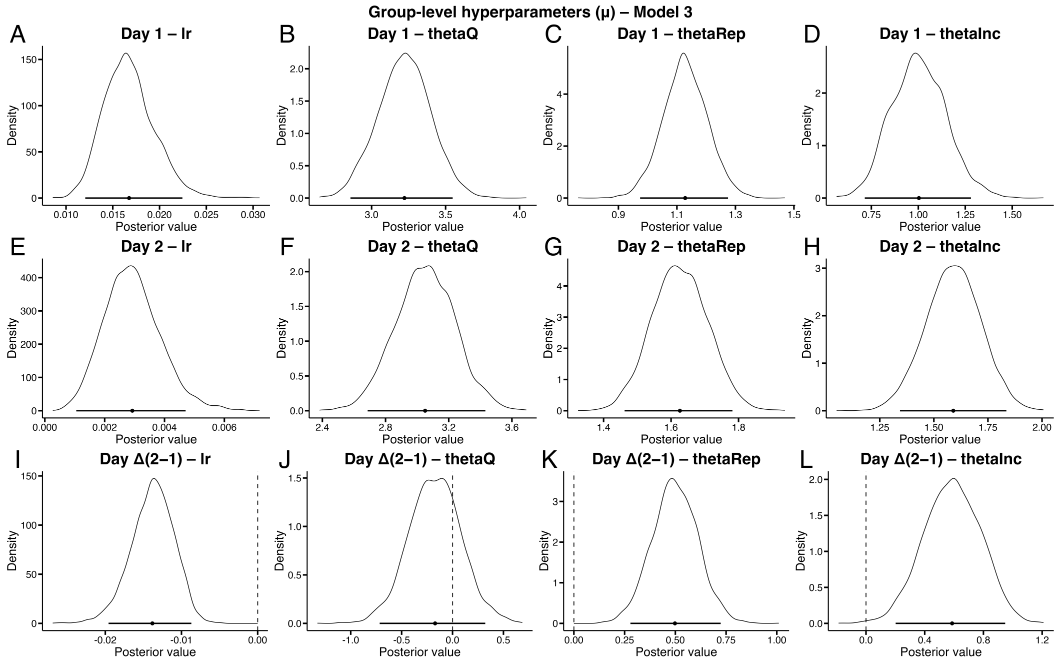

Figure S9: Hyperparameter distributions for n = 65 eligible participants: Posterior hyperparameter distributions. Top row: Hyperparameter distributions for day 1. A: learning rate (lr), B: θ_Q, C: θ_{Rep}, D: θ_{Inc}. Middle row: Corresponding posterior distributions for day 2. E: lr, F: θ_Q, G: θ_{Rep}, H: θ_{Inc}. Bottom row: Posterior difference distributions (day 2 – day 1). Positive values indicate higher parameter values on day 2

Behavioural analysis for the extended sample ($N = 65$) was performed identical to the main manuscript. A paired two-sample t-test between reaction times in STTs of the Rand and Rep conditions revealed a highly significant difference ($t(64) = 11.9$, $p < 0.0001$, 95% CI $[8.41, 11.8]$, Cohen's $d = 1.48$). Participants responded faster in the Rep condition than in the Rand condition, with an average ΔRT$= 10.1 \pm 6.8$ ms. Furthermore, a paired two-sample t-test of error rates between the Rand and Rep conditions also showed a highly significant difference ($t(64) = 6.85$, $p < 0.0001$, 95% CI $[0.52, 0.94]$, Cohen's $d = 0.85$), with fewer errors in the Rep condition and an average ΔER$= 0.73 \pm 0.86\%$.

For each DTT type (congruent, incongruent, random) we computed the proportion of optimal responses (choices of the response option with high reward probability) for each participant as the ratio of high-probability reward responses to all valid responses. A response was counted

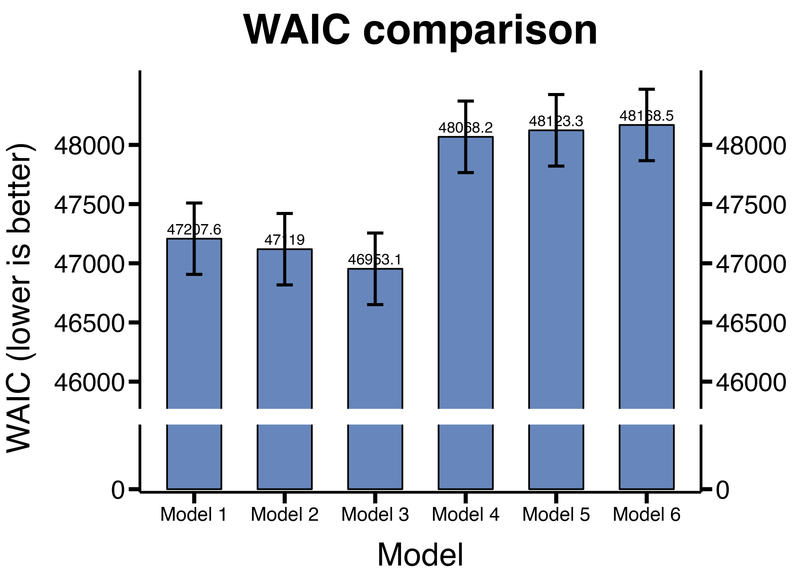

Figure S10: Model Comparison (n = 65) WAIC across both days. Models with repetition-bias (model 1 - model 3) outperform the models without repetition-bias (model 4 - model 6). Within models model 1 - model 3 error bars denote standard errors for WAIC.

as valid if participants pressed the key of one (and only one) of the presented stimuli within $600ms$. Average optimal responding across participants and all DTTs was 84.7%, showing that the extended sample was also able to do the task and generally acted in a goal-directed manner. Note that optimal responding cannot necessarily be interpreted as goal-directed responding in all DTTs: In congruent DTTs, an optimal response might arise from habitual or from goal-directed responding, while in random and incongruent DTTs, optimal responding can be considered equivalent to goal-directed responding.

A repeated-measures ANOVA was performed to analyze the effect of DTT type on optimal responding in DTTs. The results indicate a highly significant main effect of DTT type ($F(1.29, 82.33) = 65.9$, $p < 0.0001$, $\eta^2_G = 0.135$, Greenhouse-Geisser corrected). Paired t-tests of logit-transformed optimal response proportions demonstrated significant differences between all DTT types: congruent responding was significantly higher than both incongruent ($t(64) = 11.2$, $p_{adj} < 0.0001$, 95% CI $[0.76, 1.09]$) and random DTTs ($t(64) = 9.32$, $p_{adj} < 0.0001$, 95% CI $[0.46, 0.70]$), while responding in incongruent DTTs was significantly lower than in random DTTs ($t(64) = -7.28$, $p_{adj} < 0.0001$, 95% CI $[-0.44, -0.25]$). These results show that the underlying action sequence influences choice behaviour, increasing optimal responding in congruent DTTs and reducing it in incongruent DTTs, compared to random DTTs.

A repeated-measures ANOVA was next performed to analyze the effect of DTT type on reaction times. We found a highly significant main effect of DTT type ($F(1.65, 105.44) = 126.1$, $p < 0.0001$, $\eta^2_G = 0.072$, Greenhouse-Geisser corrected). Paired two-sample t-tests revealed significant pairwise differences between all DTT types: participants were faster in congruent DTTs compared to both incongruent ($t(64) = -10.7$, $p_{adj} < 0.0001$, 95% CI $[-19.4, -13.3]$ ms) and random DTTs ($t(64) = -13.6$, $p_{adj} < 0.0001$, 95% CI $[-23.5, -17.5]$ ms). Finally, reaction times in incongruent DTTs were significantly slower than in random DTTs ($t(64) = -4.07$, $p_{adj} = 0.0004$, 95% CI $[-6.08, -2.08]$ ms).

x. Post-Experiment Questionnaire

Question 1: Did you have the impression that there were phases in which the experiment appeared easier? Yes: 31 (51.67 %), No: 28 (46.67 %), Don't know: 1 (1.67 %).
Question 2: Throughout the experiment, there were phases in which a sequence of 12 button presses was repeated often. Did you notice this? Yes: 16 (26.67 %), No: 36 (60 %), Don't know: 8 (13.34 %).
Question 3: Throughout the experiment, there were phases in which a sequence of 12 button presses was repeated often. Please try to reproduce the sequence, or at least parts of it, by entering the corresponding keys (s,x,k,m) in the order of the sequence in the field below. The longest correctly reproduced sub-sequence was on average 3.8 ± 1.6 elements long. No participant was able to reproduce more than 9 correct elements of the repeating action sequence.

xi. Effects of noticing the repeating action sequence and age effects

Noticing the repeating action sequence. Of 60 participants, 16 (26.67%) reported in a post-experiment questionnaire that they had noticed a repeating sequence in the experiment, 36 (60%) participants reported that they did not notice a sequence, and 8 (13.34%) participants answered "Don't know". No participant was able to reproduce the complete action sequence. T-tests between participants who did report that they noticed and those who did not notice a sequence revealed that noticing the sequence was associated with reduced optimal responding in random DTTs ($t(50) = -2.7, p = 0.01$), and with a significantly greater difference of optimal responding between congruent and random ($t(50) = 3.5, p = 0.001$), random and incongruent DTTs ($t(50) = 2.3, p = 0.03$), and congruent and incongruent context ($t(50) = 3.4, p = 0.001$).

Age effects. As expected, higher age correlated positively with reaction times, in both DTT ($r = 0.36, p = 0.004$) and STT ($r = 0.34, p = 0.007$). Age did not correlate with error rates ($p > 0.21$). Age did also not correlate with optimal responding in any of the three DTT types ($p > 0.32$). Importantly, measures of habit-learning, such as ΔRT, ΔER, and the differences in optimal responses between the three dual-target trial types, did also not significantly correlate with age, nor show a trend. The correlation between ΔRT and the difference of optimal responses between the congruent and incongruent contexts ($C - I$) was higher in young participants than in old participants on both days, although the difference was not significant ($p = 0.1$) (median-split comparison between correlation values with two-tailed Z-test on Fisher-transformed correlation values). Participants who noticed a sequence were of similar age as those who did not (two-sample t-test: $t(50) = 1.0$, $p = 0.31$).

References

Fengler, A., Xu, P., Bera, K., Omar, A., and Frank, M. J. ("in prep"). Hssm: A generalized toolbox for hierarchical bayesian estimation of computational models in cognitive neuroscience. Manuscript in preparation.

Frölich, S., Esmeyer, M., Endrass, T., Smolka, M. N., and Kiebel, S. J. (2023). Interaction between habits as action sequences and goal-directed behavior under time pressure. *Frontiers in Neuroscience*, 16:996957.
